# Foamed glass ceramics—an upcycled scaffold for microbial biofilm development

**DOI:** 10.1007/s10529-022-03332-0

**Published:** 2022-12-12

**Authors:** Alex Kugler, Cory Trivelpiece, Robin L. Brigmon

**Affiliations:** Savannah River National Laboratory, Aiken, SC 29803, USA

**Keywords:** Microbial attachment, Circular economy, Cryopreservation, Lyophilization

## Abstract

Glass, a near infinitely recyclable material, can be upcycled to create new products such as foamed glass ceramics, which are essentially a synthetic pumice-like material. This material has been demonstrated to sustain preserved biofilms which have application in various fields based on the deployability of the product and the preserved microbes. Foamed glass ceramics have increased surface area compared to typical soda-lime glass cullet. This material has been explored for variety of applications including the growth, storage and transport of biofilms and microbial colonies which can be preserved and deployed later. Here, we demonstrate the ability for microbial cultures including BioTiger™, *Escherichia coli* K-12, *Bacillus thuringiensis*, and two environmental eukaryotic cells to colonize the upcycled glass products, undergo preservation, and regrow after 84 days of storage. The growth of preserved samples is correlated to the time spent incubating prior to preservation. These results demonstrate the applicability of this novel glass-biofilm combination in which various preserved microorganisms are able to be rapidly grown after storage on an upcycled glass product.

**Supplementary Information:**

The online version contains supplementary material available at 10.1007/s10529-022-03332-0.

## Introduction

**Fig. 1:**
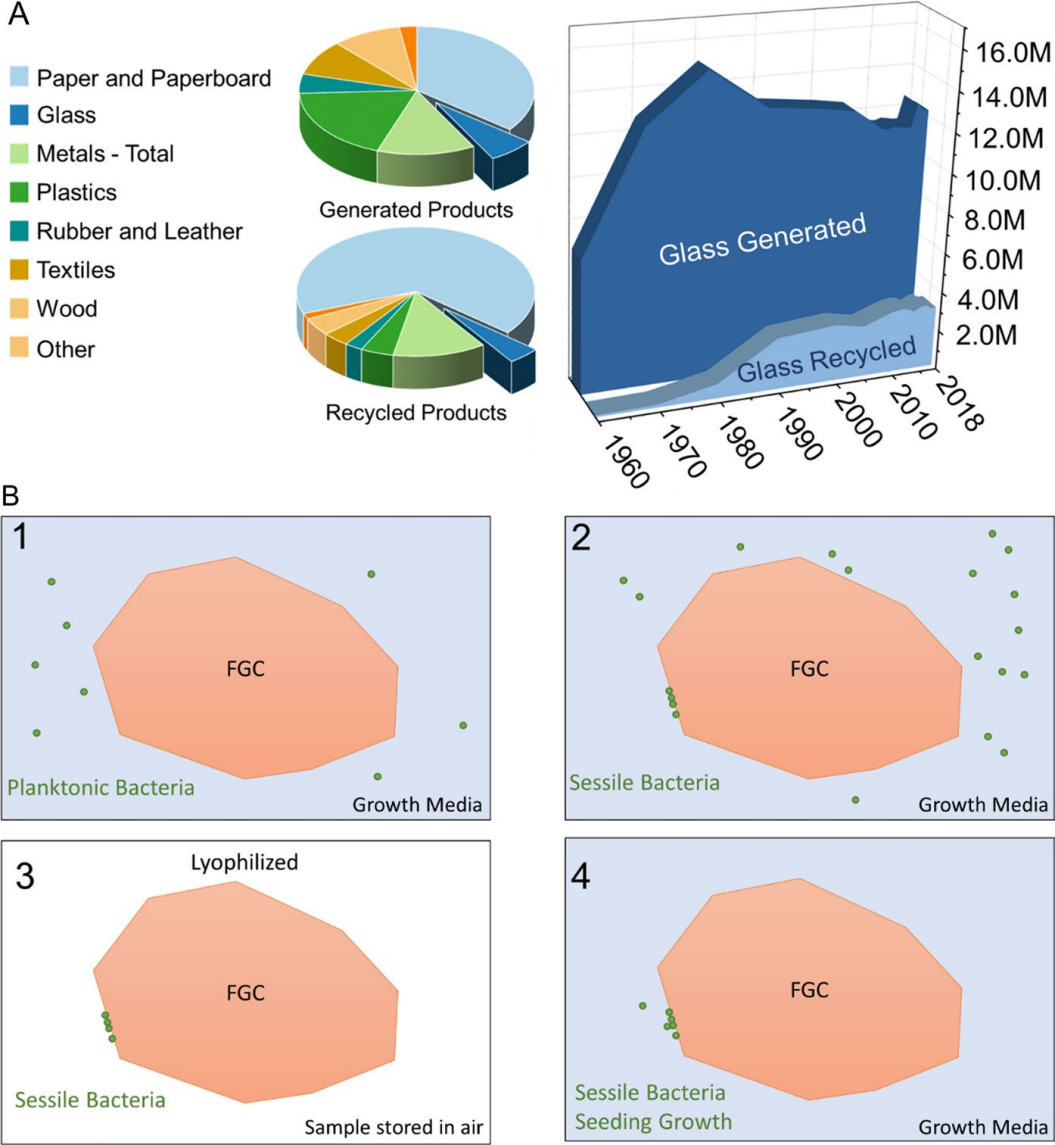
**A** shows the approximately 290 million tons of municipal solid waste and 70 million tons of recyclable products generated in the United States in 2018 the approximate percentages are shown as pie charts, demonstrating that the amount of glass recycled, and the amount of glass waste produced are nearly the same. As a function of time, glass production has greatly outpaced the tonnage recycled in the United States. In **B** the preservation process is demonstrated in four steps. Step 1 requires the bacteria to be grown in presences of the microbes which will then attach themselves to the Foamed glass ceramic (FGC) forming a biofilm or clusters of cells. After this, the FGCs are mixed with glycerol and lyophilized preserving the bacteria which can then be stored long-term before eventually being redeployed into an environment where the bacteria can regrow

Foamed glass ceramics (FGCs) are advanced, upcycled materials made from post-consumer and/or post-industrial waste glass, which is currently stockpiled in landfills worldwide. FGCs are a form of synthetic pumice that are produced at temperatures significantly lower than the liquidus temperature of the original recycled glass cullet. The result of this process is an upcycled product with significantly less environmental impact than traditional recycled products as traditional products require a full re-melt of the original glass. As of 2018, at least 75% of municipal solid waste glass generated was landfilled (Management 2018). Regardless of the amount, there is no doubt that a single stream recycling system (predominately employed in the U.S.) prevents the full economic realization of an otherwise infinitely recyclable material (Dyer 2014), see Fig. 1A. Some of this wasted economic potential has been recovered via the manufacture of traditional foam glass products; however, these materials have limited applications and account for a small fraction of the total amount of glass available for reclamation. FGCs differ from these traditional products in their ability to be economically tailored at the industrial scale to meet the demands of advanced applications.

FGCs are porous glass ceramic materials made almost entirely from waste glass or waste incinerator ashes. The composition of the final product is controlled during pre-firing batching, and the physical properties of the materials are controlled via chemical composition of the batch as well as process parameters. It is these two variables, process and composition, which allows the product to be customized and separates FGCs from traditional foam glass. Customized FGCs are being explored for use in a variety of advanced applications—one of these applications is as substrates for biofilms that can be employed in many fields including wastewater filtration, groundwater cleanup, environmental restoration, and bioremediation.

A microbial biofilm is a consortium of sessile bacteria which have established a three-dimensional community consisting of a combination of prokaryotic or eukaryotic cells embedded in a microbially produced matrix of extracellular polymeric substances (EPS) (Flemming et al. 2016). This EPS can consist of proteins, polysaccharides, humic substances, extracellular DNA, and/or additional molecules (Costa et al. 2018). Biofilms can form either on solid surfaces or form flocs which are suspended in liquid. Social and physical interactions occur intercellularly in conjunction with the EPS creating a unique and emergent lifestyle, distinctly different from that of a free-living bacterium. These unique properties of biofilms, in comparison to their free-living cellular counterparts, are well documented and can include increased antibiotic resilience (Høiby et al. 2010), increased mutations (Penesyan et al. 2019), consortia building (Flemming et al. 2016), and increased quorum-sensing-regulated mechanisms (Li and Tian 2012; Pejin et al. 2014).

Biofilms are ubiquitous throughout the environment, both natural and manmade (Flemming et al. 2016) and are imperative for almost all biogeochemical cycles in every environment including soils, water, and the subsurface (Hall-Stoodley et al. 2004). They are found on water well screens (Burkowska-But et al. 2015), on ships (Inbakandan et al. 2010), and in unconventional natural gas extraction lines (Kahrilas et al. 2015). Such formations are found throughout the environment and involve a diverse array of microorganisms. This protected mode of growth increases cell survivability and allows cells to colonize niche environments where free-living cells would struggle.

Biofilms are capable of filling technological roles and are used in water filtration systems, wastewater remediation, biocatalysts, and the generation of biofuels (Kassinger and Hoek 2020). The films often have high cell densities—10^8^–10^11^ cells per gram of wet biomass (Gebreyohannes et al. 2019). While biofilms can form from a single species, they often consist of many different species living together. This diversity aids in their survival, as well as enhances their biotechnological use by allowing them to coordinate their life cycles by staggering the expression of certain genes or proteins (Berlanga and Guerrero 2016). Biofilms also have enhanced gene exchange due to the proximity and high cell density (Madsen et al. 2012), which increases the resiliency of the bacteria compared to their free-living counterparts.

These properties increase the survivability of biofilms and allow them to grow and thrive on surfaces planktonic microbes may not have been able to colonize (Flemming et al. 2016). Considerable research has focused on biofilm growth on various surfaces such as rocks, well screens, and ships; however, these surfaces are of limited biotechnical application. Biofilms are sometimes utilized in bioreactors and various materials are used for cellular adsorption such as charcoal, vermiculite, and polypropylene (Berlanga and Guerrero 2016). These materials, unlike FGCs, are not upcycled materials and fall short when considering the importance of a circular economy. This work represents the seminal effort at growing and preserving application-specific biofilms on recycled glass products that mimic natural geological specimens, i.e., synthetic pumice. We used to upcycle, post-consumer waste glass to create an FGC-based scaffold for microorganisms with impactful potential from an environmental and industrial perspective as bioremediating strains and present a method for long-term preservation and deployment from a centralized location thereby drastically increasing the impact of this technology.

The most salient microorganism(s) we investigated in this study was BioTiger™ (BT)—a bacterial consortium developed by researchers at Savannah River National Laboratory in the 2000’s. Our intended application of this biofilm-FGC system is the remediation of hydrocarbon contamination resulting from fossil fuel extraction methods such as drilling, hydraulic fracturing, and oil sands extraction. BioTiger™, U.S. Patent 7,472,747, is a consortium of twelve aerobic bacteria isolated from a 100-year-old oil refinery in Poland that has previously demonstrated usefulness for hydrocarbon bioremediation (Brigmon et al. 2016). One particularly important feature of BT is biosurfactant production. Plaza, et al. (2007) and Reddy, et al. (2020) showed via the methylene blue active substances assay that BT produces biosurfactants. Moreover, a mixture of different biosurfactants, like those produced by the different species in BT, often presents better properties than individual biosurfactants because of a synergistic effect (Reddy et al. 2020). These biosurfactants play a critical role in the ability of the consortia to break down the hydrocarbons.

We studied the ability of BT to be directly freeze dried on FGC aggregates and investigated cell survival after rehydration by characterizing relative growth rates. Biofilms on FGCs have an advantage in bioremediation applications based on available surface area, the necessarily tortuous path induced by the connected FGC open-cell porosity, high surface area to increase the availability of environmental contaminants, deployment capacity, and the ability to incorporate beneficial nutrients into the FGC composition.

Immobilizing microorganisms has been shown, regardless of the targeted contaminant, to have increased efficiency when compared to their planktonic counterparts (Dzionek et al. 2016). This method of immobilization is similar to what is found in natural systems in the formation of biofilms; however, it is possible to immobilize microbes that do not preferentially live in a biofilm (Berillo et al. 1073). While immobilization of microorganisms can increase efficiency of the bioremediation process, the selection of a suitable substrate is critically important, especially when transitioning from lab-scale proof-of-concept to field deployable and industrially scaled technology. As such, a viable substrate must be environmentally friendly, inexpensive, and capable of supporting the microorganism. Our initial work demonstrates that BT, as well as other microorganisms, can successfully grow on, form biofilms, and be preserved for long term usage on FGCs, which can then be used to inoculate a secondary area such as a wetland, agricultural soil or water application, ocean, or wastewater processing tank, see Fig. 1B.

Cell cultures are a common part of bioremediation strategies; however, they can be difficult to deploy as the cultures can die in transport, fail to bloom, and must be chosen specifically for each environment. Here, we established the potential of FGCs to close a circular economic loop as a substrate for BT, an especially hardy biofilm-forming consortia (Brigmon et al. 2008), to grow on FGCs, tested the microcosm’s ability to be preserved for long term storage via a lyophilization process, and the resulting biofilm-FGC ability to reseed an environment.

## Materials and methods

All materials, unless otherwise stated, are of scientific grade or higher.

### Foamed glass ceramic characterization

Our initial experiments utilized an off-the-shelf Foamed glass ceramics (FGCs) provided by a commercial entity. To ascertain the basic properties of the glass the material was analyzed via powder x-ray diffraction (Analytical X^’^Pert^3^). The scan was performed from 2 to 55 degrees 2Ɵ, with a scan speed of 1 s and 0.2 step size. The surface area was analyzed via physisorption (Anton Parr autosorb IQ) using krypton gas and the Brunauer–Emmett–Teller (BET) model. Micrographs were collected to observe surface characteristics of the material with a scanning electron microscope (Zeiss Supra 40 VP, 5.1 mm working distance, 1.0 kV, high vacuum).

### Microbial cultivation

BioTiger™ bacterial consortium, *Bacillus thuringiensis (B. thuringiensis) (*ATCC 35646)*,* and *Escherichia coli* (*E. coli)* k12 (ATCC 25404), were routinely cultured on Reasoner’s 2A (R2A; Fisher Scientific) media containing the following per liter of water; 0.5 g casein acid hydrolysate, 0.5 g dextrose, 0.3 g K_2_HPO_4_, 0.024 g MgSO4, 0.5 g proteose-peptone, 0.3 g sodium pyruvate, 0.5 g soluble starch, and 0.5 g yeast extract, and was buffered to a pH of 7.2 ± 0.2 at 25 °C. Routine bacteria stocks were cultured at room temperature on a shake plate at 100 rpm. All additional experiments using bacteria were carried out using R2A media or agar prepared with 15 g/L agar (Fisher Scientific), as necessary. To test for compatibility with different cell types an environmental algal sample, maintained using Bushnell Haas broth containing the following per liter of water, 0.2 g MgSO_4_, 0.02 g CaCl_2_, 1.0 g KH_2_PO_4_, 1.0 g K_2_HPO_4_, 1.0 g NH_4_NO_3_, and 0.05 g FeCl_3_ adjusted to a final pH of 7 was used. To facilitate the measurement of BioTiger™ growth, fresh cultures were inoculated and monitored at 0, 1, 2, 3, 4, 5, 6, 8, 22, 24, 26, 28, and 48 h. At each of these time points, the optical density at 600 nm (OD_600_) was measured using a Genysis Vis20 (Thermofisher) spectrophotometer. Concurrently, samples were serially diluted, and 100 µL of the solution was plated on R2A agar in triplicate and incubated for 3 days. Dilutions without the optimal range of CFUs, 20–200, were discarded while the optimal plates were counted to correlate the optical density at 600 nm with colony forming units (CFUs; Supporting Information Fig. S1).

### Biofilm growth, attachment, and preservation

To determine the effectiveness of FGCs as a substrate for biofilm growth, 3 g of sterile, pebble sized (φ of − 3 to − 4, approximately 1 cm in diameter) FGCs were placed into 100 mL of sterile R2A media. This media was then inoculated with 1 mL of log phase cells, either BioTiger™, *B. Thuringiensis*, *E. coli* K-12*,* or the *Chlorella* spp. These initial samples were incubated at room temperature at 100 rpm for 1 week. After samples had become laden with cell mass a sterile solution of 80% glycerol was added bringing the overall concentration of glycerol to 20% and allowed to rest for 5 min. The FGCs were then removed from the solution and placed in a − 80 °C freezer (ThermoScientific). After freezing the samples were lyophilized (Labconco Benchtop 2.5 L Freeze dryer) and freeze-dried over a 48-h period. Samples were then stored in a refrigerator at 4 °C. Successful biofilm growth was determined by examining the samples using a SZX16 stereoscope (Olympus, Tokyo).

To determine the success of storage, 10 of the pebble sized treated FGCs were placed into 100 mL of R2A media 1, 3, 7, 14, 21, 28, 56, and 84 days after lyophilization. Growth was indicated by an increase in turbidity, OD_600_, over the course of 72 h at room temperature and shaking at 100 RPM.

### Regrowth

We determined the rate of regrowth and the industrial potential of preserved FGCs, by incubating a series of FGCs at 0, 2, 6, 24, 48, 72, and 196 h with BioTiger™ prior to the preservation process and then preserving as described above. Ten preserved FGCs from each incubation time were placed in 75 mL of sterile R2A broth and the OD_600_ was measured over time using a visible light spectrophotometer. This experiment was performed in duplicate.

## Results

### Materials characterization

**Fig. 2:**
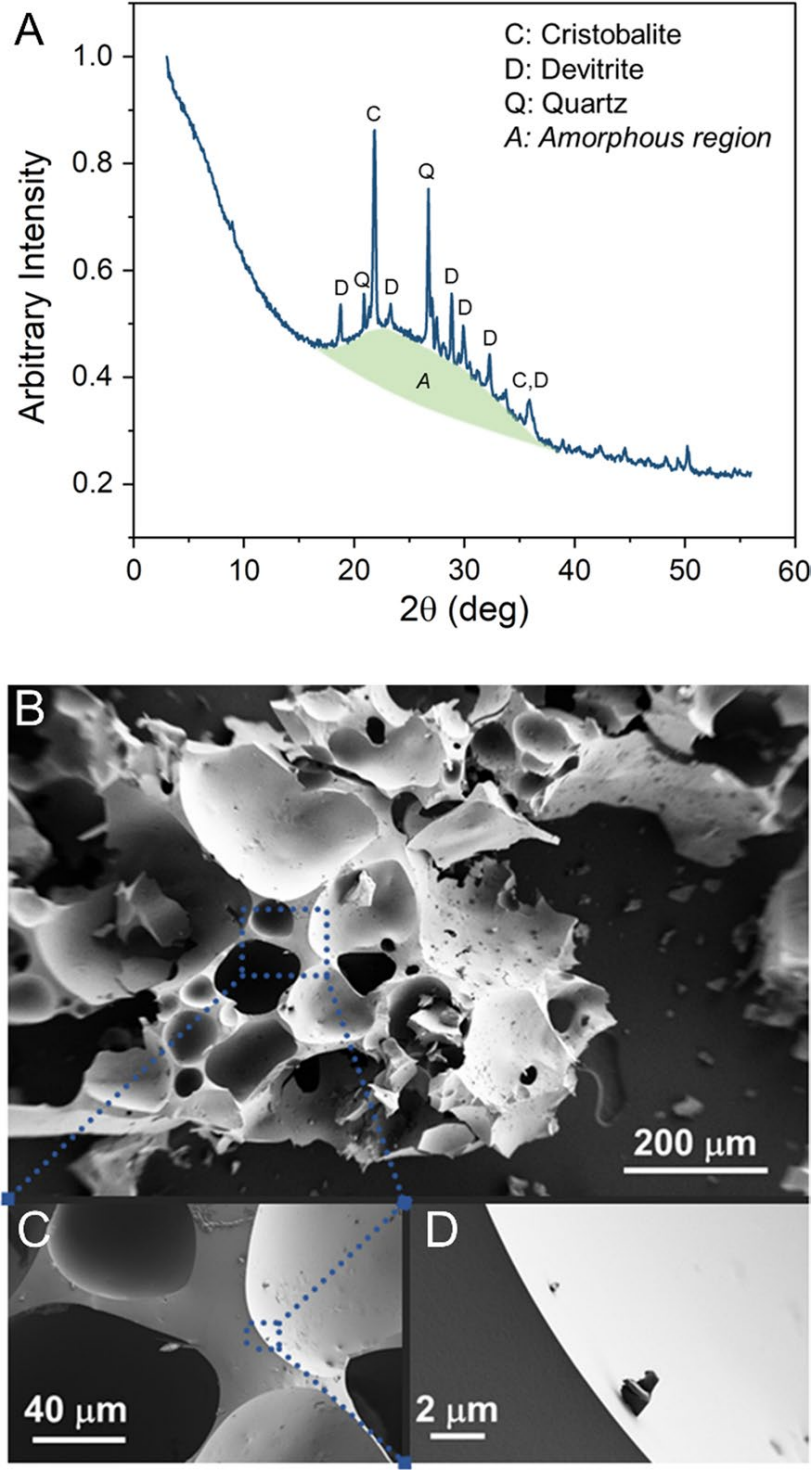
The diffractogram, **A** shows the various mineral phases in the Foamed glass ceramics (FGCs). The peaks highlighted in the figure correspond to the peaks of cristobalite, deviltries, and quartz with the characteristic amorphous hump ranging from 15 to 35 2Ɵ which is common to all amorphous glass products. A corresponding SEM micrograph, **B**-**D**, show the texture and high porosity of the FGC. Smooth edges still show some texturing and pitting which is ideal for microbial attachment

The commercial Foamed glass ceramics (FGCs) were observed to be largely amorphous (> 65%) with inclusions of cristobalite, a high temperature silica polymorph (SiO_2_), quartz (SiO_2_), and devitrite, an orthorhombic silicate (Na_2_Ca_3_Si_6_O_16_) crystallized during the cooling of the soda lime silicate-based FGC after synthesis. The X-ray diffraction pattern for this material is shown in Fig. 2A along with the main crystalline peaks and corresponding phase identification. An amorphous phase was also observed (centered on 2θ ~ 28°, FWHM ~ 10°), which is indicative of the glass from which the FGC is synthesized. The chemical composition of the FGCs was assumed to be equivalent to commercial soda-lime silicate, typical composition of this glass is 70–75 wt% SiO2, 12–16 wt% of Na2O, and 10–15 wt% CaO (Pfaender 1996)*.* The surface area of the FGC material was measured to be 0.3 m^2^/g. An SEM micrograph of the fresh FGC surface is shown in Fig. 2B–D—as can be seen in the micrographs, the surfaces are smooth, and the pore sizes range from approximately 100–500 µm. This FGC pore size is ideal for colonization of microorganisms that range in size from 1–2 µm.

### Biofilm growth, attachment, and preservation

When determining the viability of bacteria in the presence of FGCs, we opted to initially test a wide variety of bacteria, as well as several eukaryotes, to determine whether the FGCs prevented bacteria growth. While none of the tested microorganisms were negatively impacted by the presence of the FGCs, it was imperative to determine whether the preservation process left the cells viable. Each of the tested microorganisms were then regrown, checking for turbidity as a positive confirmation of successful preservation. A full list of tested microorganisms can be found in the Supporting Information Table S1. Upon completion of this initial screening, optical analyses were caried out using three of the test microorganisms, the BioTiger™ consortia, *B. thuringiensis*, and the algae.

**Fig. 3:**
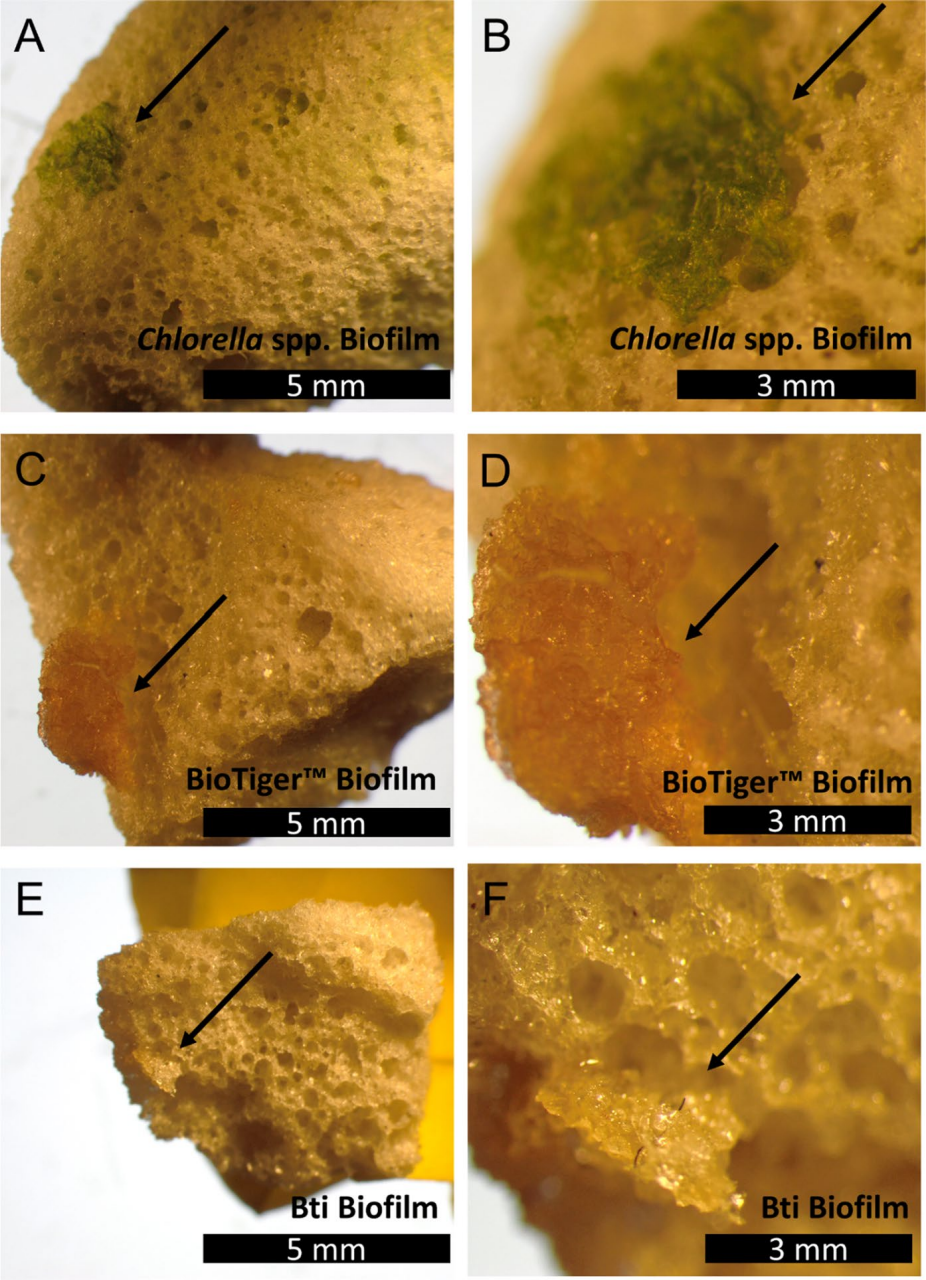
Biofilms after 1 week of incubation in the presence of Foamed glass ceramics (FGCs), persevered with glycerin and then lyophilized. The magmatic surfaces show clearly formed and preserved biofilms. Algal samples (**A** and **B**), BioTiger™ consortium (**C** and **D**) and *B. thuringiensis* (**E** and **F**) show clearly defined and lyophilized extracellular polymeric substance which has bacteria incorporated within

Preserved biofilm formations can be seen in Fig. 3. The biofilms range from 1 to 3 mm in radius and were stable in the atmosphere and under the stereoscope. The green algal cells (A and B), orange BioTiger cells (C and D) and yellow *B. thuringiensis* cells (E and F) are seen on the surface of the FGCs, attached to the porous structure of the FGC. The biofilms, which consists of cells and extracellular polymeric substances, and have been highlighted with a black arrow.

Over approximately 3 months, lyophilized FGC samples stored at 4 °C were tested for success. FGCs preserved with the BioTiger™ consortium, *B. thuringiensis*, and the algae were tested for growth after 1, 3, 7, 14, 21, 28, 56, and 84 days of storage. Each sample tested positive for an increase in turbidity in the media within 24 h for the bacterial samples and within 1 week for the algae.

### Regrowth

**Fig. 4:**
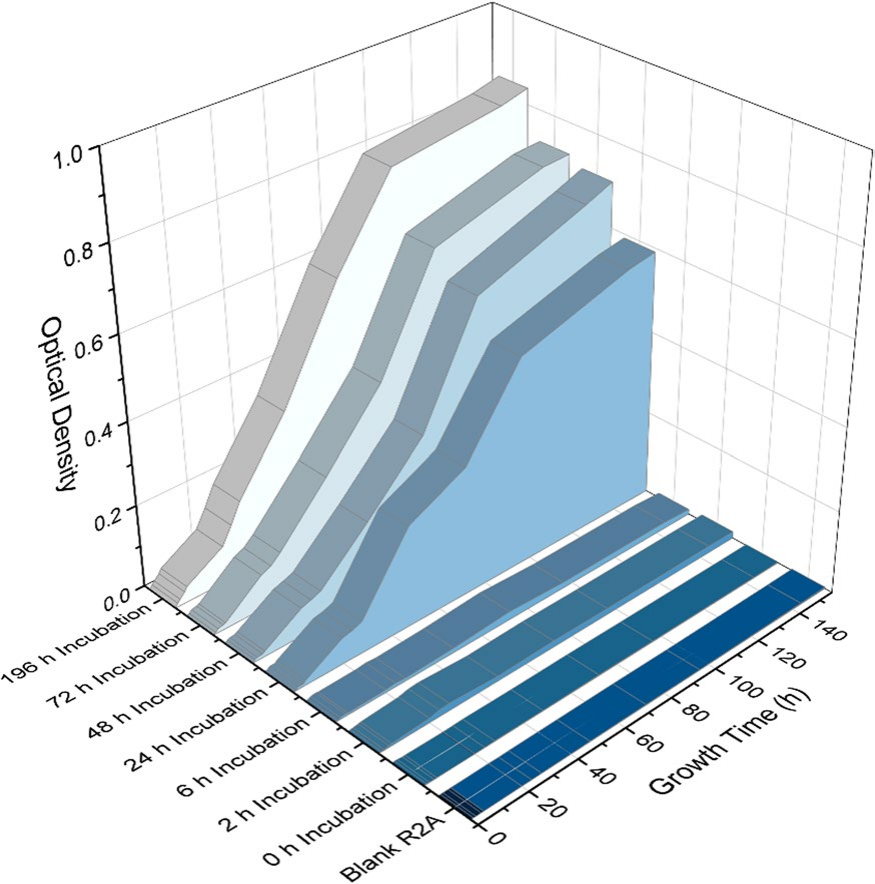
Growth curves of BioTiger™ from a preserved state on Foamed glass ceramics (FGCs). Growth occurred over several days in R2A media on a rotary shaker at 100 RPM. The growth was tracked using optical density at 600 nm. Different incubation times were tested in parallel to determine how long FGCs would need to be incubated with bacterial cultures prior to undergoing the preservation technique. This graph shows the average of duplicate experiments, error of each point is within a 5% standard deviation

The rate of regrowth from the preserved state was measured by comparing incubation times prior to preservation at 0, 2, 6, 24, 48, 72, and 196 h with BioTiger™. The results, Fig. 4. show two distinct trends amongst the samples. Samples that were incubated for 0, 2, and 6 h show no, or almost no bacterial regrowth over the incubation period of nearly a week. Samples which were incubated for 24, 48, 72, and 196 h show a linear growth trend between approximately 6 and 96 h, with an initial lag phase, which then plateaus between 100 and 140 h. Unsurprisingly, samples incubated the longest prior to preservation resulted in the fastest reestablishment of a microbial community.

## Discussion

In this study we demonstrated the ability for microbiological samples to be stored and renewed after preservation on upcycled waste glass products. These Foamed glass ceramics (FGCs) are made from a feedstock of post-consumer waste, which rather than being recycled, is unfortunately mostly discarded in landfills. The synthesis of these materials is significantly less carbon-intensive than traditional glass recycling because the process temperature is a fraction of the original glass liquidus temperature. The high surface area and relative porosity provides ample area for cells to develop biofilms which are easily preserved. The preservation process demonstrated that different lines of bacterial, algal, and fungal cells within their own biofilm could be preserved, stored, and ultimately regrown after several months of storage. The BioTiger™ consortia showed that longer incubation prior to preservation allowed the cells to populate fresh media after inoculation faster and more robustly. However, from an industrial production point of view, the difference between incubating the samples for 24 h and 196 h is inconsequential (Fig. 4). This work provides a practical application of an upcycled scaffold for microbiological preservation using a consortium of bacteria with a demonstrated scientific and industrial application of the degradation of hydrocarbons. This effort lays the groundwork for additional experimentation using both tailored FGCs for enhanced biologic activity and a host of applicable microorganisms which can be preserved, stored, and later deployed for any number of industrial, agricultural, or research applications. In particular, the demonstrated efficacy of FGCs at hosting and the regrowth of lyophilized BT establishes an industrially scalable method for deploying a circular-economic solution that will have a massive impact on remediating the environmental contamination caused by past, present and future fossil fuel extraction globally.

## Supplementary Information

Below is the link to the electronic supplementary material.

Supplementary file1 (DOCX 21 KB)
