# Supplementary material for: Foamed glass ceramics—an upcycled scaffold for microbial biofilm development
Source: Biotechnol Lett. 2022 Dec 12;45(2):225–33. doi: 10.1007/s10529-022-03332-0 (PMC9868040; doi:10.1007/s10529-022-03332-0)
Supplement: Supplementary file 1 — Supplementary file1 (DOCX 21 KB) [file 10529_2022_3332_MOESM1_ESM.docx]

**Supporting Information**

**Table S1.** List of microbial cultures which have been tested for preservation with respect to FGCs. The name, American type culture collection number (ATCC), cell type and cell type are recorded along with a positive or negative indicator for growth in conjunction with the FGCs, whether it was preserved, when it positively regrew, and whether it was imaged.

| **Table 1.** Microorganisms grown on FGCs | | | | | | |
| --- | --- | --- | --- | --- | --- | --- |
| **Name** | **ATCC** | **Cell Type** | **Growth** | **Preserved** | **Regrown** | **Imaged** |
| *Escherichia coli* K12 | 47076 | Gram - | + | + | + | - |
| BioTiger™* | N/A | Consortia | + | + | + | + |
| *Bacillus thuringiensis* | 33679 | Gram + | + | + | + | + |
| *Chlorella* spp | N/A | Eukaryotic | + | + | + | + |
| *Lecanicillium* sp | N/A | Eukaryotic | + | + | + | - |
| *Sphingomonas* sp BPH | PTA-5574 | Gram - | + | + | + | - |
| *Bacillus cereus* | 13061 | Gram + | + | + | + | - |
| *Shewanella oneidensis* | 700550 | Gram - | + | + | + | - |
| *Pseudomonas putida* | 47054 | Gram - | + | + | + | - |

***** BioTiger™ is a microbial consortium consisting of 12 microbial species.

**Figure S1.** BioTiger™ growth curves as a function of time. The optical density is plotted with corresponding CFU/mL from plates. Both optical density measurements and Cultures were grown in R2A at room temperature on a rotary shaker plate at 100 RPM. Trend line calculated from 5-24 hours show a linear growth with an R^2^ of 0.9994 and 0.9708 for the optical density and CFUs, respectively. Error bars are within the size of the symbols shown.
